# Aphid Honeydew Quality as a Food Source for Parasitoids Is Maintained in Bt Cotton

**DOI:** 10.1371/journal.pone.0107806

**Published:** 2014-09-16

**Authors:** Steffen Hagenbucher, Felix L. Wäckers, Jörg Romeis

**Affiliations:** 1 Agroscope, Institute for Sustainability Sciences (ISS), Zurich, Switzerland; 2 Centre for Sustainable Agriculture, Lancaster University, Lancaster, United Kingdom; French National Institute for Agricultural Research (INRA), France

## Abstract

Bt-transgenic cotton has proven to be highly efficient in controlling key lepidopteran pests. One concern with the deployment of Bt cotton varieties is the potential proliferation of non-target pests. We previously showed that Bt cotton contained lower concentrations of insecticidal terpenoids as a result of reduced caterpillar damage, which benefited the aphid *Aphis gossypii*. It is thus important that non-target herbivores are under biological control in Bt cotton fields. The induction or lack of induction of terpenoids could also influence the quality of aphid honeydew, an important food source for beneficial insects. We therefore screened *A. gossypii* honeydew for cotton terpenoids, that are induced by caterpillars but not the aphids. We then tested the influence of induced insect-resistance of cotton on honeydew nutritional quality for the aphid parasitoid *Lysiphlebus testaceipes* and the whitefly parasitoid *Eretmocerus eremicus*. We detected the cotton terpenoids gossypol and hemigossypolone in *A. gossypii* honeydew. Although a feeding assay demonstrated that gossypol reduced the longevity of both parasitoid species in a non-linear, dose-dependent manner, the honeydew was capable of sustaining parasitoid longevity and reproduction. The level of caterpillar damage to Bt and non-Bt cotton had no impact on the quality of honeydew for the parasitoids.These results indicate that the nutritional quality of honeydew is maintained in Bt cotton and is not influenced by induced insect resistance.

## Introduction

In 2012, insect-resistant, genetically engineered (GE) cotton expressing Cry toxins from *Bacillus thuringiensis* (Bt) was grown on 22.5 million hectares world-wide [1]. These Bt cotton plants are highly resistant to major lepidopteran pests [2], [3], and their large-scale adoption has led to area-wide population declines of some of these target pests [4], [5]. The high specificity of Cry toxins makes them a valuable tool for integrated pest management (IPM) programs as the use of Bt-transgenic varieties can lead to reductions in insecticide use with benefits for biological control [3], [6]–[8].

Research in different regions of the world has indicated that the abundance of mirid (Hemiptera: Miridae) and stink bug (Hemiptera: Pentatomidae) herbivores that are not suppressed by the insecticidal trait has increased on Bt cotton [3], [9]. The reasons for these population increases are complex and include the reduction in insecticide use and a decrease in resource competition [3], [9]–[11]. Another possible factor that could lead to the emergence of non-target pests is reduced indirect, plant-mediated competition between herbivores, which can be mediated by secondary plant metabolites [12]. Cotton plants possess a range of defence compounds that belong to a group of closely related terpenoids: gossypol, hemigossypolone, and the heliocides 1–4 [13]–[15]. The cotton defence system is inducible, and damage by some herbivores results in a systemic increase in terpenoid production [14], [16]–[19].

Because Bt cotton plants are less damaged by tissue-feeding caterpillars than conventional cotton plants [20], the overall amount of terpenoids could be lower and the plants thereby more susceptible to insects that do not induce terpenoid production and are also not affected by the Cry toxins. We previously reported that the cotton aphid *Aphis gossypii* (Glover) (Hemiptera: Aphididae) was indeed more abundant on Bt plants than on non-Bt plants infested with *Heliothis virescens* (Fabricius) (Lepidoptera: Noctuidae) larvae, and that the increase in *A. gossypii* abundance was correlated with lower levels of caterpillar-induced terpenoids [15]. We have also investigated whether this positive effect on aphids affects the next higher trophic level, i.e., aphid parasitoids. While fewer parasitoid mummies were found on caterpillar-infested non-Bt than on caterpillar-infested Bt cotton, aphid quality as hosts for *Lysiphlebus testaceipes* (Cresson) (Hymenoptera: Braconidae) was not affected by the changes in terpenoid production of the plants even though the aphids contained cotton terpenoids [21].

Adult parasitoids, however, could also be exposed to cotton terpenoids when they consume honeydew on cotton plants. Honeydew is an important carbohydrate source for parasitoids, especially in agro-ecosystems that often lack alternative sugar sources [22]–[25]. Honeydew consumption increases parasitoid longevity [24], [26], [27] and fecundity [28], and honeydew can serve as a host-finding cue for parasitoids of honeydew producers [29], [30]. However, honeydew is often inferior to nectar or nectar–sugar solutions as a food source for parasitoids [22], [24], [26], [31]–[34]. One possible reason for this is that honeydew contains insect-derived sugars that have a lower nutritional value for insects than plant-derived sugars and that may even be toxic [33], [35]. Additionally, secondary plant compounds can pass through the insect gut unaltered and can thus be present in the honeydew, which may further reduce its suitability as food [24], [34], [36]. Aphids feeding on caterpillar-infested cotton plants in which plant defenses have been induced may consume elevated levels of induced secondary metabolites. When they excrete these metabolites in their honeydew, this would in turn expose the honeydew feeding insects to higher levels of secondary metabolite in their food.

We here assessed the impact of caterpillar-induced cotton terpenoids on the quality of *A. gossypii* honeydew as a food source for two parasitoid species common in USA cotton fields, i.e., *L. testaceipes* and *Eretmocerus eremicus* (Rose and Zolnerowich) (Hymenoptera: Aphelinidae). While *L. testaceipes* is one of the principal parasitoids of *A. gossypii* [37], *E. eremicus* is an important parasitoid of *Bemisia tabaci* (Gennadius) (Hemiptera: Aleyrodidae) (Gerling et al., 2001). Although the latter species is more likely to feed on the honeydew of its host than on that of *A. gossypii*, it would still encounter aphid honeydew in its natural habitat. For example, in Tifton, GA (USA), *A. gossypii* invades cotton in early June and peaks in mid-July before populations start to collapse [38]. Aphids are, however, still present during the remaining season and often reach a second population peak in late July/August [38], which is also the time were whiteflies start to appear in the cotton fields (personal observation). In this case aphid honeydew could be an important source of nutrition when whitefly numbers are still low. Our hypothesis was that *A. gossypii* honeydew would contain lower levels of terpenoids on Bt cotton than on non-Bt cotton, such that honeydew from Bt cotton would be superior to honeydew from non-Bt cotton as a food source for the parasitoids.

## Materials and Methods

### Plant material

Two varieties of GE cotton plants provided by Monsanto Company (St. Louis, USA) were used for the experiments. One variety (“Bt cotton”), Deltapine DPL143B2 RF (event: MON15985×MON88913), expresses two Bt toxins (Cry1Ac and Cry2Ab, Bollgard II) and carries a herbicide-tolerance trait. The second variety (“non-Bt cotton”), Deltapine DPL147 RF (event: MON88913), has a similar genetic background but contains only the herbicide-tolerance trait. For insect rearing, the closely related non-GE variety Deltapine DPL491 was used. Plants were grown in 3-l plastic pots (one plant per pot) containing heat-sterilized humus-rich soil. At planting, 15 mg of the slow-release fertilizer Osmocote (16% N, 11% P_2_O_5_, 11% K_2_O; Scotts UK Professional, Bramford, UK) was added to each pot. Subsequently, the plants were fertilized weekly using 10N∶10P∶8K at 20 ml/l and were watered daily. Plants were enclosed in gauze cages (height: 71 cm, diameter: 35 cm, mesh-width: 0.264 mm) to protect them from glasshouse pests.

### Insect material

A colony of *A. gossypii* was obtained from Syngenta (Stein, Switzerland) and reared permanently on 4- to 8-week-old non-Bt cotton plants (DPL491). Larvae of *H. virescens* and adult *B. tabaci* were regularly obtained from Syngenta. For the experiments, *B. tabaci* colonies were established by enclosing five adults on the youngest fully developed leaf of a 4- to 8-week-old cotton plant (DPL 491) in a small plastic cage (diameter: 35 mm). After 2 weeks, the colonies were used for the experiments.

Mummies of *L. testaceipes* were obtained from Katz Biotech AG (Germany), and mummies of *E. eremicus* were obtained from Andermatt Biocontrol AG (Switzerland). All insects were kept at 25±5°C and 70±10% RH and under long-day conditions (16 h of light∶8 h of dark).

### Honeydew collection from induced and uninduced cotton plants

In the glasshouse, Bt and non-Bt cotton plants were used when they had four fully expanded true leaves. Plants were either infested by a single *H. virescens* larva (3^rd^ instar) that was caged on the youngest fully developed leaf for 7 days in a gauze bag or plants were left uninfested (control). *Heliothis virescens* larval weight was recorded before the larva was placed on the plant and after 7 days to calculate the weight gain.

After the larva was removed, 100 *A. gossypii* (mixed stages) were transferred to the youngest fully developed leaf of each plant (not identical to the leaf on which the larva was released). Uninfested plants were treated in the same way. The aphids were enclosed in a clip-cage built from 9-cm-diameter Petri dishes. After 7 days, honeydew collection was initiated by placing glass plates (15×15 mm) on the bottom of each Petri dish. During the following 7 days, the glass plates were collected every 24 h, and the bottom of the Petri dish was replaced to prevent growth of fungi. The honeydew-sprinkled glass plates were stored at −80°C for later use.

After the 7 days of honeydew collection, damage caused by *H. virescens* larvae was recorded. For this, the damaged leaf from each plant was collected and photographed, and the damaged leaf area was measured with ImageJ v1.42 (National Institutes of Health, USA) software. Additionally, the youngest fully developed leaf of each plant was collected for terpenoid quantification.

For analysis of the terpenoid content of honeydew, we used a different set of plants that were treated as described in the previous paragraphs. Honeydew was collect from 7–10 plants per treatment. However, collection was only conducted during the first day and not during the entire seven day period, as in the previous experiment. The glass plates used were pre-weighed before the honeydew collection. After 24 h, the plates were removed and stored at −80°C until later analysis by high-performance liquid chromatography (HPLC).

### Parasitoid longevity as affected by gossypol

Females of *L. testaceipes* and *E. eremicus* were between 1 and 14 h old when used for these experiments. To assume mating, every female parasitoid was kept in a plastic vial with one male for 4 h. Subsequently, females were kept individually in glass vials (60×11.7 mm) that were closed with Parafilm and contained a piece of cotton wool soaked with water to provide a RH>95% to minimize evaporation of water from the food sources (sucrose solutions containing gossypol and *A. gossypii* honeydew, as described in the next paragraphs). The cotton wool was replaced every other day. Survival of the parasitoids was recorded twice each day (at 9–10 am and at 5–6 pm). Experiments were conducted at 25±5°C and 85±10% RH under long-day conditions (16 h of light∶8 h of dark). Thirty females were tested per treatment and parasitoid species. Females lost or killed during handling were removed and were not included in the data analysis, leading to a total of 27–30 replications for *E. eremicus* and 28–30 replications for *L. testaceipes*.

Female parasitoids of both species where fed different concentrations of the cotton terpenoid gossypol (≥95%, Sigma, St. Louis, USA). Because gossypol is not soluble in water, it was first dissolved in 1% dimethyl sulfoxide (DMSO). Subsequently, different concentrations of gossypol (0.000001, 0.00001, 0.0001, and 0.001%) were dissolved in a 1 M sucrose solution. A 1 M sucrose solution with 1% DMSO, a pure 1 M sucrose solution, and pure water served as controls. A single drop (2 µl) of the test solution was placed on the inner surface of the Parafilm that was used to seal the vial. The Parafilm and the test solution were changed every other day.

### Parasitoid longevity as affected *A. gossypii* honeydew

Female parasitoids of both species where fed *A. gossypii* honeydew from *H. virescens*-infested and uninfested Bt and non-Bt cotton plants. For details on the experimental set-up and environmental conditions see section “Parasitoid longevity as affected by gossypol”. The following treatments were compared: water only (control), 1 M sucrose solution, honeydew from uninfested Bt cotton, honeydew from infested Bt cotton, honeydew from uninfested non-Bt cotton, and honeydew from infested non-Bt cotton. Thirty females were tested per treatment and parasitoid species. Females lost or killed during handling were removed and were not included in the data analysis, leading to a total of 24–30 replications for *E. eremicus* and 27–30 replications for *L. testaceipes*.

Honeydew was provided *ad libitum* on the glass plates, on which it was collected and was replaced daily. The plates were carefully broken in two pieces to fit them into the vial, one halve per vial. The wet cotton wool was replaced every other day.

### Parasitoid reproductive capacity as affected by *A. gossypii* honeydew

The parasitoids *L. testaceipes* and *E. eremicus* were prepared and fed with honeydew as described for the longevity experiment. After 24 h of feeding, each *L. testaceipes* female was removed from the glass tube and placed in a Petri dish (diameter: 50 mm) containing parts of a non-Bt (DPL491) cotton leaf with >100 *A. gossypii* of mixed stages. After 4 h, the parasitoid was removed, and the cotton leaf was placed on a non-transgenic cotton seedlings to allow the aphids to settle on the plant. The plants were checked daily for parasitoid mummies. Mummies were collected, placed in plastic containers (17×17×8 mm), and checked daily for emerging parasitoids. The number of mummies produced per female, parasitoid emergence rate, and the sex ratio of parasitoid offspring were recorded.

After 2, 4, and 6 days of feeding on honeydew, each *E. eremicus* female was removed from the glass tube and placed in a plastic container (17×17 mm). The container was placed over a colony of *B. tabaci* nymphs on a non-Bt cotton seedling (see section “Insect material” for details). After 4 h, the container was removed and the parasitoid was transferred back into the glass tube. The number of parasitoids emerging from parasitized *B. tabaci* was recorded.

Both experiments were conducted at 25±5°C and 75±10% RH. In the case of *E. eretmocerus* each treatment was represented by 23–26 parasitoid females. For *L. testaceipes* there were 26–31 females per treatment.

### Parasitoid gustatory response to gossypol

Starved, water-satiated female parasitoids (≤14 h old, mated) were used for this experiment. To ensure that females were water-satiated, they were individually kept in a plastic vial (17×17×8 mm) containing a piece of cotton wool soaked with water for a minimum of 1 h before they were used in the experiment. At the start of the experiment, a droplet of the test solution (1 µl for *L. testaceipes* and 0.3 µl for *E. eremicus*) was placed on the lid of the plastic vial. The parasitoid's tendency to walk upwards soon brought it into contact with the test solution. The parasitoids were observed until they made contact with the droplet, which happened within 60 sec in all cases. After the parasitoid contacted the test solution, its behavior was recorded. The reaction was either scored as acceptance (feeding for a minimum of 5 s) or rejection (less than 5 s of contact). When the test solution caused a feeding response, the duration of the feeding event (time during which the mouthparts were in contact with the test solution) was recorded. Observations were made with a dissecting microscope.

The following test solutions were used: 1 M sucrose solution, 1 M sucrose+1% DMSO, and sucrose/DMSO solutions containing 0.000001, 0.00001, 0.0001, and 0.001% gossypol. In total, 28–33 parasitoids of each species were observed per treatment.

### HPLC analyses of terpenoids in plant material and honeydew

Pre-weighed honeydew-covered glass plates (15×15 mm), obtained as described in the “Honeydew collection” section, were placed in a Petri dish that contained a water-saturated piece of cotton wool. The Petri dishes were closed with Parafilm and kept in a climatic chamber (25°C and 85% RH) for 30 min to saturate the honeydew with water. Subsequently, each glass plate was weighed on a microbalance (Mettler Toledo MX5; division d = 1 µg; tolerance ±2 µg). To adjust for the weight loss of honeydew through evaporation of water during the measurement, the weight of the samples was recorded after 10 s on the balance.

Samples were placed in a 200-ml Erlenmeyer flask, and 5 ml of water was added. The flasks were shaken for 30 minutes (in darkness) to dissolve the honeydew in the water, and the solution was transferred into 10-ml plastic tubes. To extract the gossypol from the solution, 1 ml of heptane was added, and the heptane phase was transferred to a new vial. This was repeated three times. Heptane was evaporated with compressed air at 50°C. The gossypol residue was redissolved in 100 µl of acetonitirile (Multisolvent HPLC grade, Scharlau, Sentmenat, Spain), water (purified by a Gradient A10, Millipore, Billerica, USA), and 85% phosphoric acid (Fluka, Buchs, Switzerland) (80∶20∶0.1) and used for HPLC. A 50-µl volume of each sample was injected into the HPLC.

Terpenoid content (gossypol, hemigossypolone, heliocides 1 and 4, and heliocides 2 and 3) was analyzed by HPLC using the protocol previously described in detail [15]. Because the terpenoid concentrations in the leaves were high, 1000 µl of the extraction solution was used, and only 10 µl was injected into the HPLC.

### Statistical analysis

All analyses were conducted using R 2.13.2.

Plant damage and weight gain by *H. virescens* were analyzed using the Welch *t*-test for non-homogenous variances. This test was chosen after detecting a non-homogenous variance using Levene's Test for Homogeneity of Variance. The effect of gossypol on the acceptance rate of sucrose solution by parasitoids was analyzed by comparing all treatments against DMSO using Pearson's Chi-squared test. Significance levels were adjusted with Bonferroni-correction, resulting in an adjusted α = 0.01.

One-way analyses of variance (ANOVA) was used to analyze data sets with homogenous variance (tested with Levene's Test for Homogeneity of Variance) and normal distribution (analyzes using Q-Q Plots). This was the case for the terpenoid contents of plants and of honeydew, the effect of gossypol on the feeding time of parasitoids, longevity of both parasitoids and the assessed parameters of the *L. testaceipes* reproductive capacity after honeydew feeding (number of mummies, offspring, emergence rate, and the sex ratio). Some data sets were transformed to meet ANOVA requirements (log transformation: terpenoids content of plants and of honeydew, feeding time on gossypol; arc sin transformation: emergence rate and sex ratio after honeydew feeding). Means were subsequently separated using the Tukey HSD-test. Data from parasitoids that were provided only with water were omitted from the analysis, to reduce Heterogeneity of Variance.

The total number of offspring produced by *E. eremicus* females after honeydew feeding was analyzed using Kruskal-Wallis rank sum test.

## Results

### *Heliothis virescens* performance, plant damage, and terpenoid concentration in leaves

When infested with a single *H. virescens* larva for 7 days, Bt cotton plants remained nearly undamaged (mean ± SE of the area consumed on the infested leaf: 0.1±0.03 cm^2^) compared to non-Bt cotton plants (38.3±5.01 cm^2^; *t*-test: t = −7.52; *P*<0.001). No larva on Bt cotton (n = 26) was alive after 7 days, but all larvae on the non-Bt cotton survived and gained considerable weight (mean ± SE weight gain per larva: 177.7±20.22 mg; n = 26).

The constitutive expression of foliar terpenoids in the youngest fully developed leaf did not differ between uninfested Bt and non-Bt cotton plants. Total terpenoid levels were significantly increased in the youngest leaves of herbivore-infested non-Bt plants 7 days after caterpillar damage had ceased (ANOVA, *F*_3,78_ = 19.5, *P*<0.001) (Table 1). The terpenoid concentration increased 186% relative to the concentration in uninfested non-Bt cotton. The concentration of all terpenoids except hemigossypolone increased significantly in response to herbivore damage. Terpenoid levels did not differ between infested and uninfested Bt cotton (Table 1).

**Table 1:** Terpenoid concentration (ng/mg dw) in leaves of Bt and non-Bt cotton as affected by prior infestation with *Heliothis virescens* in a glasshouse experiment.

Terpenoids	ANOVA			Concentration (ng/mg dw ± SE)			
	df	*F*	*P*	Bt uninfested	Non-Bt uninfested	Bt infested	Non-Bt infested
**HGQ**	3,78	0.57	0.64	4078±374.6 a	4105±410.1 a	4615±443.7 a	6936±587.1 a
**G**	3,78	21.6	<0.001	1353±212.2 b	1685±173.6 b	2141±307.6 b	5774±697.8 a
**H1/H4**	3,78	12.5	<0.001	335±27.4 b	314±28.7 b	296±23.4 b	591±46.6 a
**H2/H3**	3,78	10.4	<0.001	702±52.4 bc	612±60.2 c	964±74.6 ab	1303.7±161.2 a
**Total**	3,78	19.6	<0.001	6470±567.6 b	6717±581.5 b	8118±745.3 b	12614±1541.6 a

Terpenoid concentration (mean ± SE; n = 10) was measured in the youngest fully developed leaf of each Bt and non-Bt cotton plant that was uninfested or infested with *H. virescens* (3^rd^ instar) for 7 days. HGQ: hemigossypolone; G: gossypol; H1/H4: heliocide 1 and 4; H2/H3: heliocide 2 and 3.

Means in a row followed by different letters are significantly different (*P*≤0.05; Tukey HSD test).

### Terpenoid concentration in *A. gossypii* honeydew

Gossypol and hemigossypolone were detected in the honeydew of *A. gossypii* feeding on cotton (Table 2). Terpenoid concentrations did not differ between the honeydew produced by aphids fed on Bt and non-Bt cotton plants or between *H. virescens*-infested and uninfested plants (ANOVA, *F*_3,32_ = 2.02, *P* = 0.13). Terpenoid concentration in honeydew was <1% of that in the leaf tissue.

**Table 2:** Terpenoid concentrations (ng/mg fw) in the honeydew of *Aphis gossypii* feeding on cotton.

Terpenoids	ANOVA			Concentration (ng/mg fw)			
	df	*F*	*P*	Bt uninfested	Non-Bt uninfested	Bt infested	Non-Bt infested
**HGQ**	3,17	0.38	0.77	10.4±4.90	30.3±13.33	12.6±4.31	39.2±16.58
**G**	3,26	1.1	0.37	4.2±1.07	7.4±1.72	6.3±2.13	40.4±33.75
**H1/H4**	ND	ND	ND	ND	ND	ND	ND
**H2/H3**	ND	ND	ND	ND	ND	ND	ND
**Total**	3,32	2.02	0.13	11.1±3.37	35.9±13.40	15.4±3.71	71.6±37.76

Values are means ± SE, n = 7–10. Honeydew was collected from aphids on Bt and non-Bt cotton plants that were uninfested or infested with larvae of *Heliothis virescens* (3^rd^ instar) for 7 days. HGQ: hemigossypolone; G: gossypol; H1/H4: heliocide 1 and 4; H2/H3: heliocide 2 and 3.

ND- not detectable.

### Parasitoid longevity as affected by gossypol and *A. gossypii* honeydew

**Figure 1:**
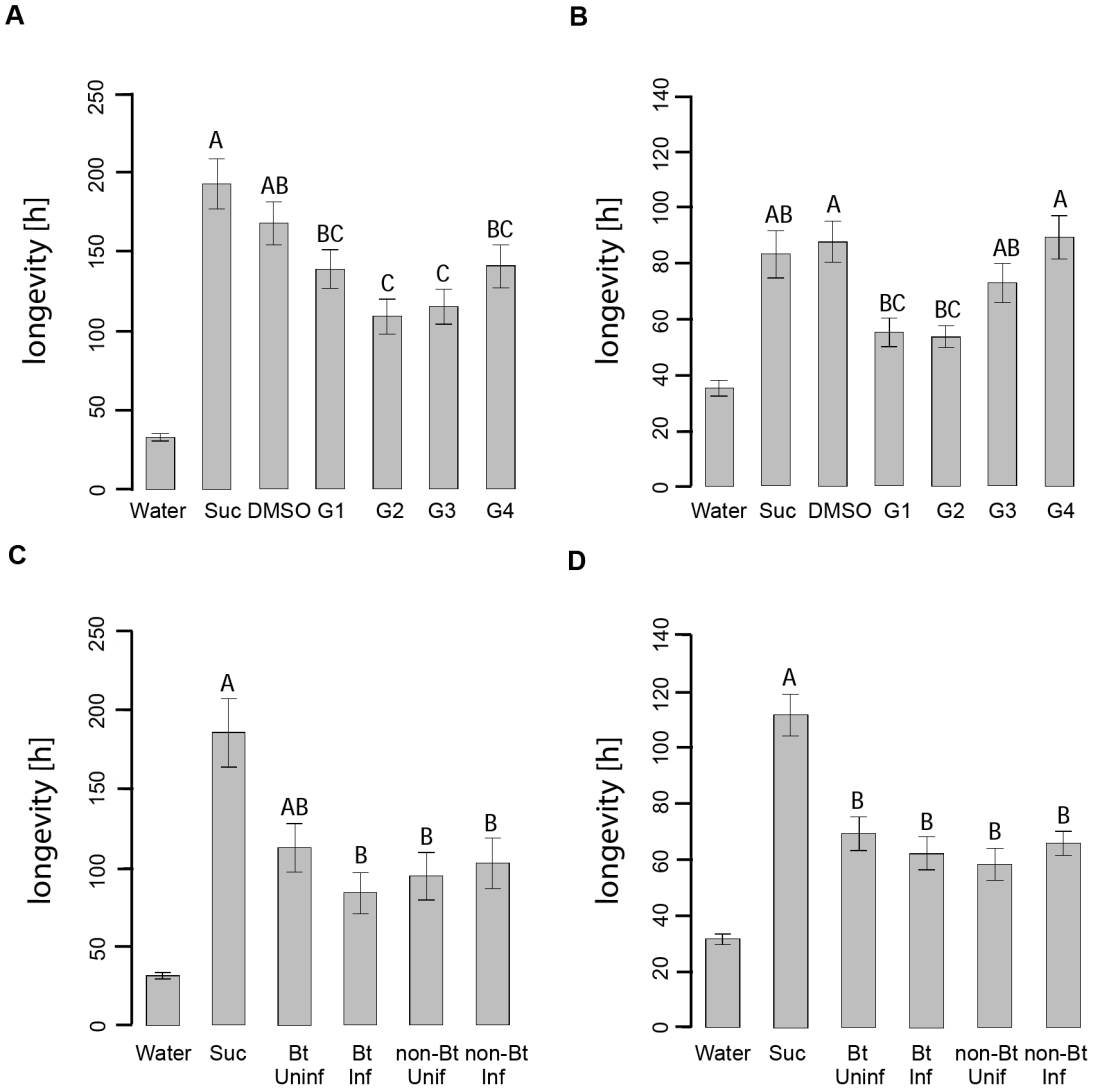
Parasitoid longevity when fed with gossypol or honeydew from *Aphis gossypii*. Longevity (hours ± SE) of (A) female *Eretmocerus eremicus* (n = 27–30) and (B) female *Lysiphlebus testaceipes* (n = 28–30) when fed with: Water, 1 M sucrose solution (Suc), 1 M sucrose+1% DMSO (DMSO), or sucrose/DMSO solutions containing different concentrations of gossypol (G1: 0.000001% gossypol; G2: 0.00001%, G3: 0.0001%; G4: 0.001%). Longevity (hours ± SE) of (C) female *Eretmocerus eremicus* (n = 24–30) and (D) female *Lysiphlebus testaceipes* (n = 27–30) when fed with: Water, 1 M sucrose solution (Suc), or honeydew from *Aphis gossypii* that were kept on Bt or non-Bt cotton that were uninfested (Uninf) or infested (Inf) with a *Heliothis virescens* larva. Within each panel, different letters above bars indicate statistically significant differences (Tukey HSD test, *P*<0.05). The water control was not included in the statistical analysis.

The longevity of *E. eremicus* females significantly differed when the females were fed with sucrose, sucrose+DMSO, or sucrose+DMSO+gossypol solutions (ANOVA; *F*_5,164_ = 6.00, *P*<0.001) (Figure 1A). *Eretmocerus eremicus* longevity was significantly shorter with gossypol at 0.00001 and 0.0001% than with the DMSO control solution. Feeding on gossypol at a lower (0.000001%) and a higher (0.001%) concentration did not significantly affect *E. eremicus* longevity. The longevity of *L. testaceipes* females was also significantly affected by feeding on the gossypol and control solutions (ANOVA, *F*_5,170_ = 4.76; p<0.001) (Figure 1B). *Lysiphlebus testaceipes* longevity was significantly shorter with gossypol at 0.000001 and 0.00001% than with the DMSO control solution (Tukey HSD test, p<0.05), but was unaffected by higher gossypol concentrations. For both parasitoid species, longevity was similar with the DMSO control solution and the pure sucrose solution (p>0.05).

### Parasitoid longevity as affected by *A. gossypii* honeydew

*Eretmocerus eremicus* longevity significantly differed between females fed with honeydew from different sources and the sucrose control solution (ANOVA, *F*_4,128_ = 4.28; *P*<0.001) (Figure 1C). Parasitoid longevity was shorter when the females were fed with honeydew rather than with the sucrose solution, except when the honeydew was obtained from uninfested Bt cotton. Among the honeydew treatments, *E. eremicus* longevity did not differ significantly among honeydews collected from Bt or non-Bt cotton plants that were infested or uninfested with an *H. virescens* larva. Similarly, the longevity of *L. testaceipes* females was shorter when the females were fed with *A. gossypii* honeydew rather than with the sucrose control solution (Figure 1D), but longevity did not differ among the four honeydew treatments (ANOVA, *F*_4,138_ = 13.04; *P*<0.001).

### Parasitoid reproductive capacity as affected by *A. gossypii* honeydew

The food source did not affect the number of mummies produced, the number of emerged adults, adult emergence rate (%), or the progeny sex ratio for *L. testaceipes* (Table 3). The reproductive capacity of *E. eremicus* females on days 2, 4, and 6 after feeding on honeydew was low (it ranged from 1.2±0.25 to 1.9±0.45 progeny per female) and did not differ among food treatments (Kruskal-Wallis rank sum test: df = 4, χ^2^ = 1.87; *P* = 0.76).

**Table 3:** *Lysiphlebus testaceipes* reproduction as affected by *Aphis gossypii* honeydew.

Food source	N	No. of mummies produced per plant	No. of emerged adults	Emergence rate (%)	Females (%)
**1 M Sucrose**	27	16.4±3.32	9.3±2.03	56.6	52.4
**Bt unifested**	26	14.6±2.88	6.8±1.32	46.2	54.6
**Bt infested**	30	13.9±2.69	6.7±1.21	48.5	51.7
**non-Bt uninfested**	31	19.0±2.75	9.1±0.63	47.5	50.2
**non-Bt infested**	27	18.8±3.56	9.2±1.53	48.7	53.6
**ANOVA**					
**df**		4,137	4,137	4,134	4,114
***F***		0.63	0.72	0.52	0.53
*P*		0.64	0.58	0.72	0.71

Values are means ± SE, n = 26–31. Female parasitoids were fed for 24 h with 1 M sucrose or with honeydew produced by *Aphis gossypii* feeding on Bt and non-Bt cotton plants that were uninfested or infested with larvae of *Heliothis virescens* (3^rd^ instar). The females were then allowed to parasitize *Aphis gossypii* for 4 h.

### Parasitoid gustatory response to gossypol

The percentage of *L. testaceipes* females that accepted the test solutions ranged from 61.3 to 84.4% and did not differ between the gossypol treatments and the DMSO control (Pearson's χ^2^-test with Bonferroni-correction: *P*>0.01). The average (± SE) feeding time ranged from 25.5±3.8 to 32.4±5.53 s and did not significantly differ among the treatments (ANOVA, *F*_5,130_ = 0.79, *P* = 0.56).

The percentage of *E. eremicus* females that accepted the test solutions ranged from 78.6 to 100% and did not differ between the gossypol treatments and the DMSO control (Pearson's χ^2^-test with Bonferroni-correction: *P*>0.01). The average feeding time ranged from 57.3±9.07 to 89.8±14.93 s and did not significantly differ among the treatments (ANOVA, *F*_5,156_ = 0.64, *P* = 0.67).

## Discussion

Gossypol and other cotton terpenoids that are produced as a response to caterpillar damage are ingested by *A. gossypii* and excreted in honeydew. Because Bt cotton suffers much less caterpillar damage than non-Bt cotton, we expected that the terpenoid concentration in *A. gossypii* honeydew would be lower on Bt cotton than on non-Bt cotton and that parasitoid fitness might be greater when feeding on honeydew from Bt cotton. Although gossypol at specific concentrations reduced the longevity of two hymenopteran parasitoid species parasitoid fitness was unaffected by honeydew source.

### Gossypol concentration in honeydew

Phloem-feeders that consume insecticidal. plant compounds must protect themselves against these toxic compounds. Certain species like *Brevicoryne brassicae* (L.) (Hemiptera: Aphididae) are known to detoxify or sequester secondary plant compounds [39], [40]. Besides metabolizing a toxin, herbivores can excrete it either with their feces, or in the case of phloem-feeders, in their honeydew. The glucosinolate sinigrin was found in the honeydew of *Myzus persicae* (Sulzer) (Hemiptera: Aphididae) feeding on *Brassica nigra* (L.) (Brassicales: Brassicaceae) [41], cardenolides were present in *Aphis nerii* (Boyer de Fonscolombe) (Hemiptera: Aphididae) honeydew from milkweed [42], and alkaloids have been reported from honeydew of various species including *Aphis jacobaeae* (Schrank) (Hemiptera: Aphididae) feeding on milkweed [36], [43].

Although cotton terpenoids have been detected in the feces of *H. virescens* [44], the current report provides the first evidence for the presence of terpenoids, i.e., gossypol and hemigossypolone, in the honeydew of a phloem-feeder. The heliocides 1–4 were not detected. The latter result may be attributed to the fact that heliocides 1–4 occur only at relatively low levels in the plant or that they are digested in the aphid gut. Compared to the concentrations of secondary metabolites in the honeydew of other plant–aphid systems, the concentrations of cotton terpenoids in *A. gossypii* honeydew were very low. Based on honeydew fresh weight, concentrations of cotton terpenoids in honeydew never exceeded 0.01% and were usually closer to 0.001%. While we could detect the terpenoids, the quantification of such low amounts reached the limits of our analytical methods (as is indicated by the high variability of terpenoid concentrations) and thus needs to be taken with caution. Although our obtained data suggest an increased concentration of terpenoids in herbivore-damaged non-Bt cotton, this could not be supported by the statistical analysis. For comparison, previous studies reported cardenolide concentrations as high as 46% in the honeydew of *A. nerii* [42] and pyrrolizidine alkaloid concentrations as high as 0.76% of in the honeydew of *A. jacobaeae* [43]. The data are, however, not directly comparable because the latter two studies presented the toxin concentration based on honeydew dry weight. The relatively low concentration of cotton terpenoids in honeydew in the present study may be explained by an efficient detoxification by the cotton aphids. Alternatively, the concentration of terpenoids in phloem sap may be low, given their hydrophobic properties.

### Impact of gossypol on parasitoids

While the impact of gossypol on lepidopteran herbivores is well known [14], [45], little information is available on its effect on other arthropods and especially on predators and parasitoids. We found that certain gossypol concentrations reduced the longevity of females of the parasitoids *L. testaceipes* and *E. eremicus*. The effect was non-linear in that the maximum effect occurred at intermediate (*E. eremicus*) or low (*L. testaceipes*) concentrations. This lack of a linear relation between gossypol dose and parasitoid longevity cannot be explained by parasitoid deterrence at high gossypol concentrations because no such deterrence was evident in our gustatory response experiment. A similar lack of a linear dose-response was detected with female spined soldier bugs *Podisus nigrispinus* (Dallas) (Hemiptera: Pentatomidae) [46]. The results of earlier tri-trophic studies were mixed. The fitness of the parasitoid *Campoletis sonorensis* (Cameron) (Hymenoptera: Ichneumonidae) was reduced when its hosts had fed on diets with high concentrations of gossypol but was enhanced when its hosts fed on diets with low concentrations of gossypol [47]. In contrast, the aphid parasitoid *Lysiphlebus japonica* (Stary & Schlinger) (Hymenoptera: Braconidae) did not develop differently in hosts from plants with high or low gossypol concentrations [48]. In our previous study, *L. testaceipes* fitness was similar when its hosts developed on caterpillar-induced and uninduced cotton plants [21].

### Impact of honeydew on parasitoids

Although the parasitoids consumed *A. gossypii* honeydew, their longevity was much shorter when fed on honeydew rather than on a sucrose solution. The aphid host plant, i.e., Bt or non-Bt plants that were infested or uninfested with a *H. virescens*, did not influence honeydew quality in terms of parasitoid fitness. The reproductive capacity did not differ between parasitoids that had fed on honeydew compared to those that had fed on sucrose, and again, the response to honeydew was not influenced by plant type (Bt or non-Bt) or prior exposure to a chewing herbivore (a caterpillar). The observed fecundity of *E. eremicus* was relatively low, which was most likely caused by the short exposure time of the parasitoid to its host. For example, Soler & van Lenteren [49] reported a total fecundity for this species of around 160 eggs per female over a 14 day lifespan, corresponding to about 10 eggs per day. This ratio compares well with the observed fecundity of *L. testaceipes* which has been reported to be about double to that of *E. eremicus* in a much shorter life-time [50].

We thus conclude that neither the genetic transformation nor the induced resistance of Bt cotton against caterpillar damage greatly affects honeydew quality. The overall concentration of cotton terpenoids in honeydew was probably too low to affect the parasitoids. Furthermore, the ingestion of the terpenoids by *A. gossypii* apparently did not result in a change in honeydew composition. A change in honeydew sugar and amino acid composition has been documented as a response to the ingestion of *Galanthus nivalis* agglutinin by *Rhopalosiphum padi* (L.) (Hemiptera: Aphididae), and such changes affected the nutritional quality of the food source for the aphid parasitoid *Aphidius ervi* (Haliday) (Hymenoptera: Braconide) [34]. In the case of *E. eremicus*, it should be noted that *Eretmocerus* species are host feeders [51]. It is possible that females fed on their host during parasitization and thereby obtained an alternate source of energy that might have countered any negative effects of the honeydew diet on parasitoid reproductive capacity. Whether gossypol or other terpenoids are present in the hemolymph of the hosts is unknown.

## Conclusions

Honeydew from *A. gossypii* feeding on cotton can be utilized as a carbohydrate source by the parasitoids *L. testaceipes* and *E. eremicus*. While cotton terpenoids were detected in the honeydew, the presence of these insecticidal compounds did not adversely affect the adult parasitoids. Overall, honeydew quality as a food source did not differ between Bt-transgenic and non-transgenic cotton plants. Similarly, it was not altered when plants had been attacked by caterpillars and terpenoid production was induced. Consequently, honeydew quality and utilization by natural enemies is unlikely to differ between Bt and non-Bt cotton fields.
